# Effects of Precooling on Endurance Exercise Performance in the Heat: A Systematic Review and Meta-Analysis of Randomized Controlled Trials

**DOI:** 10.3390/nu16234217

**Published:** 2024-12-06

**Authors:** Laikang Yu, Zhizhou Chen, Weiliang Wu, Xinhao Xu, Yuanyuan Lv, Cui Li

**Affiliations:** 1 Beijing Key Laboratory of Sports Performance and Skill Assessment, Beijing Sport University, Beijing 100084, China; sunflowerlyy@bsu.edu.cn; 2 Department of Strength and Conditioning Assessment and Monitoring, Beijing Sport University, Beijing 100084, China; 730415czz@sina.com (Z.C.); wwwuweiliang@163.com (W.W.); x15711324733@163.com (X.X.); 3 China Institute of Sport and Health Science, Beijing Sport University, Beijing 100084, China; 4 School of Physical Education (Main Campus), Zhengzhou University, Zhengzhou 450001, China; 5 School of Basic Medical Sciences, Zhengzhou University, Zhengzhou 450001, China

**Keywords:** precooling, endurance exercise performance, time trial, time to exhaustion

## Abstract

An increasing number of studies have explored the effects of precooling on endurance exercise performance in the heat, yet the available results remain inconsistent. Therefore, this study aimed to investigate the effects of different precooling strategies on endurance exercise performance in the heat. A comprehensive search was conducted across PubMed, Web of Science, Cochrane, Scopus, and EBSCO database. The Cochrane risk assessment tool was employed to evaluate the methodological quality of the included studies. A meta-analysis was subsequently conducted to quantify the standardized mean difference (SMD) and 95% confidence interval for the effects of precooling on endurance exercise performance in the heat. Out of the initially identified 6982 search records, 15 studies were deemed eligible for meta-analysis. Our results showed that precooling significantly improved time trial (TT) performance (SMD, −0.37, *p* < 0.01, *I*^2^ = 0%) and time to exhaustion (TTE) performance in the heat (SMD, 0.73, *p* < 0.01, *I*^2^ = 50%). Further subgroup analyses revealed that external precooling is more effective in improving TT performance (SMD, −0.43, *p* = 0.004, *I*^2^ = 0%) and TTE performance (SMD, 1.01, *p* < 0.001, *I*^2^ = 48%), particularly in running-based performances (TT, SMD, −0.41, *p* = 0.02, *I*^2^ = 0%; TTE, SMD, 0.85, *p* = 0.0001, *I*^2^ = 31%). Precooling is an effective approach to improve endurance exercise performance in the heat. External precooling is more effective in improving endurance exercise performance, particularly in running-based performance.

## 1. Introduction

A decrease in endurance exercise capacity has been reported when exercising in hot environmental conditions compared to normal and cold environmental conditions [1]. During exercise, high temperature can produce excessive heat stress on the human body, and a series of stress reactions occur, such as increased core temperature, accelerated metabolism, elevated body temperature, and increased sweating with impaired evaporation [2], which is mainly manifested in the advancement of fatigue and a reduction in power output. It has also been pointed out that high temperature can increase the muscle temperature of athletes, leading to a decrease in neuromuscular function and affecting sports performance [3].

Previous studies have shown that the rapid increase in core temperature is the primary factor affecting endurance exercise performance when exercising in the heat [4], and there is a core temperature threshold [5]. When the core temperature rises to reach the core temperature threshold, it will reduce the excitability of the central nervous system and cardiovascular function, leading to fatigue [6]. Typically, the endurance exercise performance [7] and intermittent exercise performance [8] deteriorate when the core temperature approaches approximately 40 °C, especially in prolonged endurance activities [9].

The current hypothesis suggests that the critical limiting factor for endurance exercise performance in the heat is elevated core temperature [10]. It has been suggested that precooling in the heat decreases the initial core temperature of the athlete and increases the margin between the initial core temperature and the temperature, which affect sports performance, thereby improving endurance exercise performance. The proposal and evaluation of numerous precooling strategies have been prompted by the hypothesized connection between elevated core temperature and diminished endurance exercise performance.

A growing body of research has examined the effects of precooling on endurance exercise performance in the heat, while findings of available studies were conflicting. Previous studies have shown that both internal and external precooling is effective in lowering core temperature, thereby enhancing endurance exercise performance [11,12]. In addition, a meta-analysis indicated that pre-cooling can improve subsequent intermittent and prolonged sports performance in the heat [13]. However, some studies have shown that precooling is effective in lowering body temperature, but is ineffective for endurance exercise performance. For example, Levels et al. [14] and Stevens et al. [15] showed that ice slurry ingestion reduced core temperature but was ineffective for endurance exercise performance. This may be due to the differences in precooling strategies, endurance performance tasks, and types of endurance exercise test.

Two previous meta-analyses have investigated the effects of precooling on endurance exercise performance in the heat. However, one of these meta-analyses also included studies that applied a cooling intervention during exercise [16], which may have influenced their findings regarding the precooling intervention. In addition, the other meta-analysis incorporated studies where the experiments were conducted at temperatures below 26 °C [17], potentially impacting their conclusions about applying precooling interventions in the heat. Therefore, we conducted a comprehensive systematic review and meta-analysis of randomized controlled trials (RCTs) to explore the effects of different precooling strategies on endurance exercise performance in the heat. In this study, we exclusively included studies that applied a cooling intervention prior to exercise and conducted experiments at temperatures above 26 °C [9].

## 2. Materials and Methods

### 2.1. Design

This study was conducted in accordance with the Preferred Reporting Items for Systematic Review and Meta-Analysis (PRISMA) guidelines [18]. The protocol has been registered with PROSPERO (CRD42023448784).

### 2.2. Search Strategy

We searched the PubMed, Web of Science, Cochrane, Scopus, and EBSCO databases from the inception dates to 6 May 2024, using the following keywords and MESH terms: (1) exercise, exercising, endurance, performance, pace, pacing, sport, sports, sporting, aerobic; (2) precool, pre-cool, pre-cooling, precooling, pre-cooled, precooled, cool, cooled. We also manually searched references listed in the identified systematic reviews and meta-analyses. Two authors independently completed the article screening using a standardized form.

### 2.3. Eligibility Criteria

Inclusion criteria were (1) RCTs; (2) inclusion of a precooling group and control group; (3) experiments conducted at >26 °C [9]; (4) studies using endurance exercise performance as the outcome measure.

Exclusion criteria were (1) publications that were not in English (such as Korean, Chinese, Japanese, French, German, Russian, Spanish, etc.); (2) studies conducted on animals; (3) reviews articles; and (4) conference papers.

### 2.4. Data Extraction

Two authors independently performed the data extraction, mainly including (1) study characteristics (first author’s surname, publication year); (2) intervention characteristics (precooling strategies, environment conditions, endurance exercise test protocols, types of endurance exercise); (3) subject characteristics (*n*, gender, age); (4) outcome characteristics (endurance exercise performance).

### 2.5. Methodological Quality Assessment

The methodological quality of the included studies was evaluated using the Cochrane risk of bias tool, which was based on selection bias, performance bias, detection bias, attrition bias, reporting bias, and other biases. Two authors independently conducted the methodological quality assessment, and disagreements were resolved by discussing with a third author. We evaluated the certainty of the cumulative evidence using the Grading of Recommendations, Assessment, Development, and Evaluations (GRADE) system. Within this framework, the certainty of the evidence was categorized as high, moderate, low, or very low. Our assessment took into account factors such as risk of bias, inconsistency, indirectness, imprecision, and publication bias.

### 2.6. Statistical Analysis

We extracted the mean and standard deviation (SD) values pertaining to endurance exercise performance in both the precooling and control groups. For studies reporting standard error (SE) and 95% confidence interval (CI), we calculated the SD according to previous studies [19,20]. Data were polled using fixed- or random-effects models to determine the standardized mean difference (SMD) and 95% CI. The *I*^2^ static was used to assess heterogeneity, where *I*^2^ < 25%, 25 < *I*^2^ < 75%, *I*^2^ > 75% indicate low, moderate, and high heterogeneity, respectively [21,22]. If there was a high heterogeneity (*I*^2^ > 60%), sensitivity analysis and subgroup analysis were used to interpret the results [23].

For subgroup analyses, we tried to investigate the effects of precooling strategies, endurance performance tasks, and types of endurance exercise test on endurance exercise performance in the heat. The forest plots were generated using RevMan 5.4 software (Cochrane, London, UK), and sensitivity analysis and funnel plot were performed using Stata 17.0 software (Stata Corp, College Station, TX, USA). Statistical significance was considered for outcomes with a *p* < 0.05.

## 3. Results

### 3.1. Studies Selection

**Figure 1:**
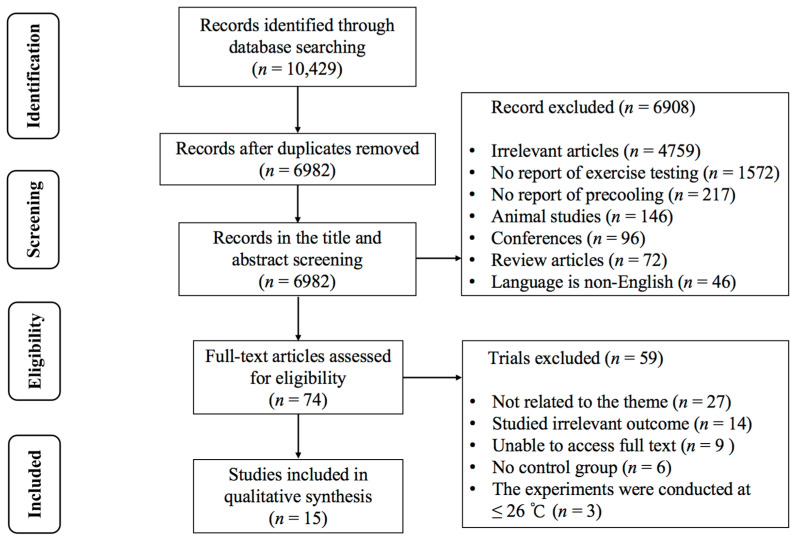
PRISMA flowchart of study selection.

Figure 1 illustrates the initial retrieval of 10,429 records from the databases. A totalof 6982 studies remained after excluding duplicates and 74 studies remained after the title and abstract screening. Upon reading the full text, 59 studies were excluded for the following reasons: (1) not related to the theme (*n* = 27); (2) studied irrelevant outcome (*n* = 14); (3) unable to access full text (*n* = 9); (4) no control group (*n* = 6); (5) the experiments were conducted at ≤26 °C (*n* = 3). Finally, 15 studies [6,24,25,26,27,28,29,30,31,32,33,34,35,36,37] met the inclusion criteria.

### 3.2. Characteristics of the Included Studies

Table S1 presents the characteristics of precooling interventions and participants. Among the included studies, there were 236 participants in the 24 precooling groups and 175 participants in the 15 control groups. Of the fifteen studies, ten studies [6,24,25,27,28,30,31,32,36,37] provided data for time trial (TT) and five studies [26,29,33,34,35] provided data for time to exhaustion (TTE). In addition, five studies [28,29,31,34,35] used internal precooling and eleven studies [6,25,26,27,29,30,32,33,34,36,37] used external precooling. Furthermore, an endurance exercise performance test was conducted on a treadmill in eight studies [25,26,28,29,30,33,36,37] and on a bicycle in six studies [6,24,31,32,34,35].

### 3.3. Meta-Analysis

**Figure 2:**
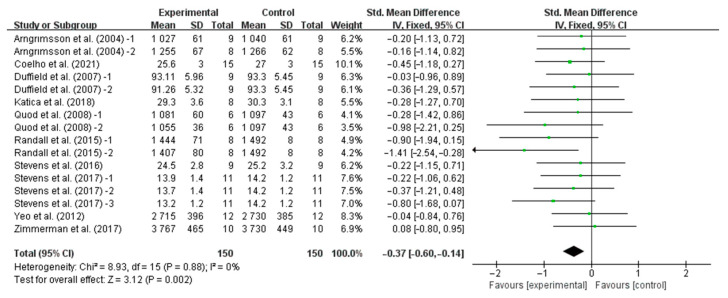
Meta-analysis results of the effects of precooling on TT performance in the heat [6,24,25,27,28,30,31,32,36,37].

**Figure 3:**
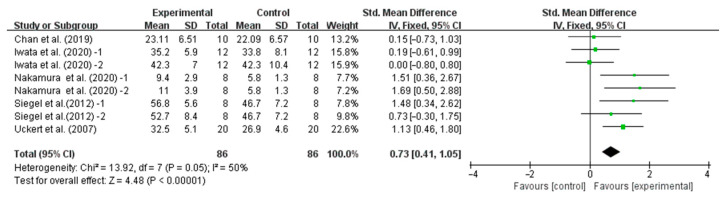
Meta-analysis results of the effect of precooling on TTE performance in the heat [26,29,33,34,35].

Our results showed that precooling had a significant effect on improving TT performance [SMD, −0.37; 95% CI, −0.60 to −0.14, *p* = 0.002, *I*^2^ = 0%, Figure 2] and TTE performance [SMD, 0.73; 95%CI, 0.41 to 1.05, *p* = 0.00001, *I*^2^ = 50%, Figure 3].

### 3.4. Subgroup Analysis

#### 3.4.1. TT Performance

**Figure 4:**
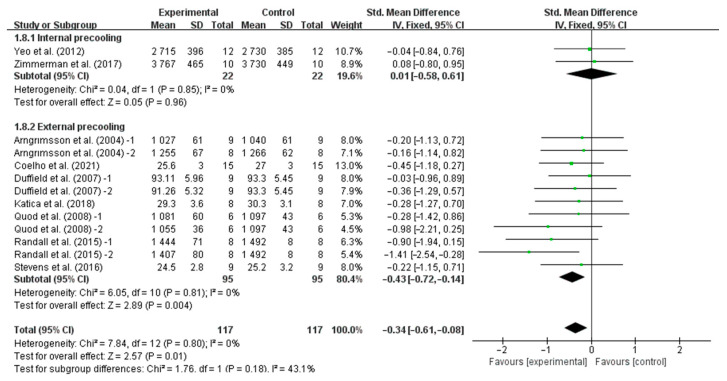
Meta-analysis results of the effects of different precooling strategies on TT performance in the heat [6,25,27,28,30,31,32,36,37].

Stratifying the analysis by precooling strategies, external precooling significantly improved TT performance (SMD, −0.43, 95% CI, −0.72 to −0.14, *p* = 0.004, *I*^2^ = 0%), while internal precooling did not significantly improve TT performance (SMD, 0.01, 95% CI, −0.58 to 0.61, *p* = 0.96, *I*^2^ = 0%, Figure 4).

**Figure 5:**
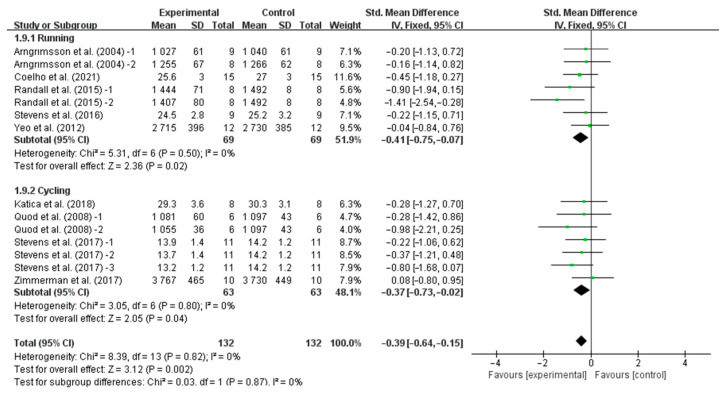
Meta-analysis results of the effects of precooling on running- and cycling-based TT performance in the heat [6,24,25,28,30,31,32,36,37].

In addition, when analyzing the subgroup based on endurance performance tasks, precooling was found to significantly improve TT performance as measured by running (SMD, −0.41, 95% CI, −0.75 to −0.07, *p* = 0.02, *I*^2^ = 0%) and cycling (SMD, −0.37, 95% CI, −0.73 to −0.02, *p* = 0.04, *I*^2^ = 0%, Figure 5), with running-based TT performance exhibiting a more pronounced effect.

#### 3.4.2. TTE Performance

**Figure 6:**
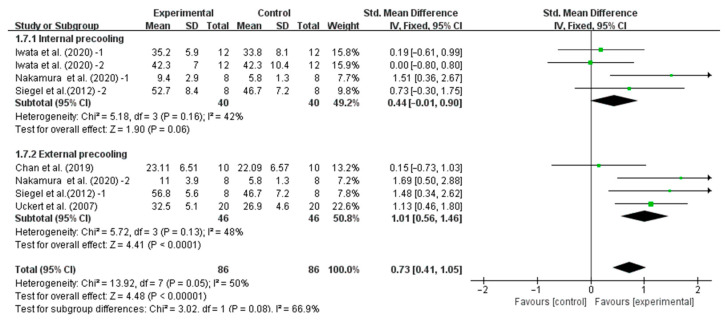
Meta-analysis results of the effect of different precooling strategies on TTE performance in the heat [26,29,33,34,35].

Stratifying the analysis by precooling strategies, external precooling significantly improved TTE performance (SMD, 1.01, 95% CI, 0.56 to 1.46, *p* < 0.0001, *I*^2^ = 48%), while internal precooling did not significantly improve TTE performance (SMD, 0.44, 95% CI, −0.01 to 0.90, *p* = 0.06, *I*^2^ = 42%, Figure 6).

**Figure 7:**
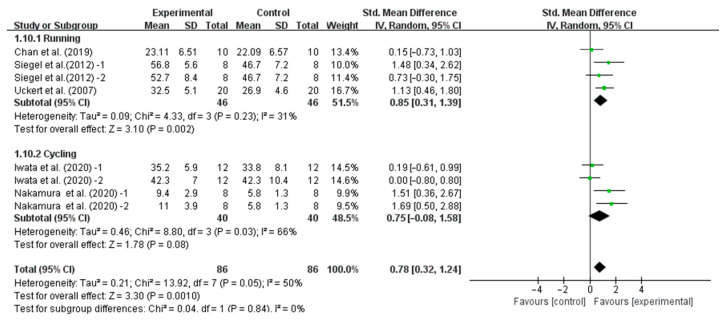
Meta-analysis results of the effect of precooling on running- and cycling-based TTE performance in the heat [26,29,33,34,35].

In addition, when analyzing the subgroup based on endurance performance tasks, precooling was found to significantly improve TTE performance as measured by running (SMD, 0.85, 95% CI, 0.31 to 1.39, *p* = 0.002, *I*^2^ = 31%), while precooling did not significantly improve TTE performance as measured by cycling (SMD, 0.75, 95% CI, −0.08 to 1.58, *p* = 0.08, *I*^2^ = 66%, Figure 7).

### 3.5. Risk of Bias

We used the Cochrane risk assessment tool to assess the quality of the included studies in terms of biases such as selection, performance, detection, attrition, reporting, and others (Figure S1). The quality was scored according to three levels: low risk, high risk, and unclear. The observed asymmetry in funnel plots indicated the presence of publication bias (Figure S2). As shown in Table S3, we evaluated two evidence syntheses using the GRADE approach. The certainty of the TTE results was rated as moderate, primarily due to potential publication bias, resulting in the downgrading of evidence quality. The TT results were assessed as having low certainty, with the quality downgraded mainly because of a high risk of detection bias, significant statistical heterogeneity, and potential publication bias in the two included studies.

### 3.6. Sensitivity Analysis

Sensitivity analysis indicated that the positive effects of precooling on TT and TTE performances in the heat remained stable and consistent in both direction and magnitude, regardless of the exclusion of any individual study (Figures S3 and S4).

## 4. Discussion

The study aimed to explore the effects of different precooling strategies on endurance exercise performance in the heat. A total of 15 studies were included, with the results conclusively demonstrating that precooling had a significant effect on improving TT and TTE performances in the heat. Further subgroup analyses revealed that external precooling is more effective in improving TT and TTE performances, particularly in running-based performances.

### 4.1. Effects of Precooling on Endurance Exercise Performance in the Heat

This study demonstrated that precooling holds the potential to improve endurance exercise performance in the heat, as indicated by improvements in TT and TTE performance. Our findings align with previous studies, which reported that consuming 1–1.6 L of cold drinks prior to exercise led to an increase in exercise capacity [38]. Athletes who wore an ice singlet for 30 min of precooling before exercise experienced a significantly longer exhaustion time during testing [33]. However, the precise mechanism underlying the beneficial effects of precooling interventions on sports performance in the heat have not been fully elucidated.

Firstly, it is crucial to comprehend the mechanisms by which high-temperature environments induce fatigue impair physical performance. Key factors influencing exercise performance include environmental temperature, relative humidity, an athlete’s ability to regulate core temperature, as well as the intensity and duration of exercise [39]. Studies have highlighted that exposure to high temperature can lead to a decline in neuromuscular function and movement ability [40].

Central fatigue is believed to predominantly affect the function of the central nervous center within the central nervous system. Research into the impact of high temperatures on the central nervous system mainly focuses on cognitive function, sensory response, and neural activation. Within the nervous system, elevated muscle temperature can regulate contractile properties, oxidative capacity, and nutrient substrate utilization. Notably, alternations in muscle temperature directly affect muscle contraction via the nervous system, thus affecting sports performance [41]. For every 1 °C change in muscle temperature, muscle contraction performance changes by 2 to 5% [42]. Compared to moderate temperature, athletes in high-temperature environments experience a faster decline in muscle capacity as exercise intensity increases [43]. A meta-analysis has shown that precooling is a common method employed by athletes to reduce perceived stress in high-temperature environments [15]. In addition, studies on ice slurry intake have reported that consuming ice slurry during rest periods before exercise slows the rise in thermal sensation (TS) during subsequent TTE tests [44]. Furthermore, several studies have demonstrated that ingesting menthol or ice plasma can positively influence the rating of perceived exertion (RPE) and TS, delay the rapid decline in cognitive function and perceptual response, improve the stability of the central nervous system, and contribute to endurance exercise performance in high-temperature environments [45,46]. With the implementation of external precooling strategies, such as applying menthol or low-temperature clothing to the skin surface, positive effects on RPE, TS, and endurance exercise performance have been observed [17,47].

Secondly, the hypothalamus serves as a crucial temperature sensor in the human body, controlling the sending of inhibitory signals during exercise and regulating the selection of exercise intensity [48]. High-temperature environments can influence the hypothalamus to issue premature inhibitory signals, which can be mitigated through precooling before significant brain and muscle dysfunction occurs, thereby reducing central fatigue [49]. Previous studies have shown that precooling strategies can effectively improve athletes’ RPE and thermal comfort (TC) during exercise, helping athletes relax their central nervous system, slow down the rate of inhibitory signal transmission from the brain center, and enhance endurance exercise performance [32]. Additionally, Xue et al. [50] found that although precooling measures did not significantly alter sweat rate or body temperature, they had a positive impact on athletes’ TC, and the results indicated improved endurance exercise performance. Furthermore, research has demonstrated that TC is fully reduced early in endurance exercise following precooling, and the exhaustion time of the precooling group was significantly longer than that of the non-precooling group in TTE tests. The authors speculated that the precooling strategy fully reduces the stress on the central nervous system, reducing functional consumption and improving endurance exercise performance [34]. Moreover, research has also shown that precooling alters the sensory feedback of the thermoregulatory system, mitigating the overheating protection mechanism of the central nervous system [51], allowing athletes to perform at a higher level for longer periods in high-temperature environments. Increasingly, studies are demonstrating that precooling can reduce the impact of high temperature on the central nervous system and improve athletes’ heat storage capacity, effectively improving exercise endurance performance [52].

Body temperature comprises core temperature and peripheral temperature [53]. Core temperature mainly reflects the temperature of vital internal organs within the human body, which can be measured through various internal bodily locations [54]. The peripheral temperature is predominantly composed of skin temperature, subcutaneous tissue temperature, and skeletal muscle temperature [55]. Additionally, variations in both core and skin temperatures are primarily influenced by the external environment’s temperature and humidity [56,57]. Research has shown that an elevation in core temperature is the primary factor affecting exercise ability in high-temperature environments [58]. Furthermore, studies have also discovered a critical threshold for core temperature during exercise in the heat [3]. When the core temperature reaches 40 °C, the muscular power output diminishes as the central nervous system initiates adjustments to prevent further rises in core temperature and the corresponding decline in muscle power output [59]. As core temperature rises, neural regions of the brain responsible for movement are inhibited [60], exacerbating fatigue or early termination of exercise due to skeletal muscle exertion and sensory feedback from the cardiovascular system [61]. Moreover, alternation in core temperature and skin temperature can significantly impact an athlete’s perception ability [62]. Flouris et al. [63] found that when core temperature rapidly increases due to exercise in a high-temperature environment, athletes’ blood pressure and heart rate surge, while their cycling power output decreases.

By using precooling strategies to reduce core temperature before exercise, athletes can increase their body’s heat storage capacity [64,65], enabling them to perform more work before reaching a critical core temperature, thereby delaying fatigue and improving endurance exercise performance [63]. The primary effect of the precooling strategy is, therefore, to reduce and stabilize the rise in core and skin temperatures in high-temperature environments [66]. Several studies have shown that without warm-up, the use of precooling strategies can effectively lower core temperature and improve sports performance [67]. Tyler et al. [15] also noted that facial precooling can reduce brain temperature and delay the rise in core temperature, mitigating the negative impact of high temperature on athletes and improving endurance exercise performance. In addition, precooling moderates the increase in core or skin temperature during the warm-up phase, mediated by its effect on RPE [68]. For instance, Riera et al. [69] found that body temperature decreased, RPE diminished towards the latter stages of exercise following precooling, and improved endurance exercise performance was observed at the end. Concurrently, several studies have indicated that the precooling strategy effectively reduces core temperature and aids in maintaining athletes’ cognitive ability during exercise [32], while a gradual rise in core temperature during exercise is beneficial for athletes to sustain exercise intensity [70].

Furthermore, athletes must attend to the heat balance of their bodies when exercising in high-temperature environments. Only when the heat production and heat dissipation are in balance can optimal sports performance be achieved [71]. When high ambient temperatures are similar to skin temperature, the heat from the peripheral temperature of the human body must be dissipated through sweating to maintain heat balance [72]. However, during exercise, the human endocrine system may struggle to keep up with the body’s heat dissipation demands [73], leading to continuous heat accumulation inside the human body and rapid increase in core and skin temperatures [73]. Watkins et al. [74] reported that precooling reduces heat storage before exercise, improves athletes’ heat storage capacity, delays fatigue onset, and enhances endurance exercise performance. Moreover, Ross et al. [75] pointed out that the precooling strategy effectively manages athletes’ temperature gradients during exercise, minimizing internal heat loss and maximizing their endurance ability.

During exercise, the central vascular system must fulfill the dual requirements of body temperature regulation and metabolism. When exercising in a high-temperature environment, the heart rate surges rapidly, potentially reaching physiological thresholds or causing exhaustion [76]. Simultaneously, this heightened heart rate shortens the cardiac cycle, resulting in a decrease in cardiac filling, end-diastolic volume, and stroke volume, which in turn reduces cardiac output, mean arterial pressure, and blood flow to skeletal muscle [77,78]. Additionally, as core body temperature rises, reduced circulating blood flow diminishes cardiac filling pressure, while the accelerated heart rate curtails the filling period, leading to a decrease in cerebral blood volume [77], significantly impacting endurance exercise performance. Numerous studies have reported that precooling strategies can mitigate muscle tension stemming from peripheral vascular contraction caused by elevated skin temperature [79], with a primary indicator being increased venous blood flow within the cardiovascular system [80]. Following precooling in a high-temperature environment, the heart rate diminishes, and stroke volume augments during the initial exercise phase, indicating that precooling enhances blood supply during exercise and bolsters endurance exercise performance [81]. Choo et al. [82] suggested that the heightened stroke volume might stem from augmented venous return due to diminished skin blood flow and improved myocardial contractility resulting from core temperature and sympathetic activation.

Furthermore, Siegel et al. [83] emphasized that precooling strategies can mitigate the heat stress response while concurrently reducing heart rate. Post-precooling and cooling, the exercise-induced heart rate diminishes, stroke volume intensifies, and central blood supply is effectively bolstered.

### 4.2. Subgroup Analysis

Our subgroup analysis revealed that precooling is more effective in improving running-based TT and TTE performances in the heat. For instance, Stanley et al. [84] reported that men cycling after adopting an internal precooling strategy involving ice slurry intake prior to a 30 min ride showed no improvement in endurance exercise performance. In contrast, Ross et al. [75] found that cold water immersion, an external precooling strategy, reduced skin temperature by 4.4 °C in cyclists and led to a 6% increase in subsequent endurance exercise performance. Additionally, Siegel et al. [29] compared the effects of internal and external precooling and concluded that external precooling was more conducive to endurance exercise performance. This suggests that the precooling strategy is a crucial factor. Previous studies showed that external precooling may more effectively regulate body temperature, control metabolism and heat dissipation, thereby enhancing cognitive function and self-regulation ability in high-temperature environments, leading to improved endurance exercise performance [26,36,37].

The successful implementation of an external precooling strategy to improve endurance exercise performance is attributed to its ability to effectively balance the body’s heat conduction, heat convection, and nutrient metabolism [85]. In addition, external precooling mainly acts on the body surface temperature during temperature control, thus preventing excessive heat from evaporating into the environment in hot conditions [86].

The external precooling strategies included in this meta-analysis were head precooling, cold water immersion, and ice clothing. Studies on cold water immersion have found that this method can effectively reduce skin temperature, core temperature, and average body temperature prior to exercise [87], thereby controlling athletes’ heat balance and increasing their running distance during endurance exercises lasting over 30 min [88]. Its function is mainly manifested in the soaking of the skin surface in cold water, as a significantly reduced skin temperature enhances athletes’ heat storage capacity [89], delays the increase in core temperature, and reduces sweat secretion during exercise. Importantly, the effect of the cold water immersion strategy is consistent with the cooling requirements for endurance exercise in a high-temperature environment [90]. In practice, a cold water immersion program lasting up to 30 min may be more effective than the traditional 60 min program [91]. All studies included in this meta-analysis that utilized the cold water immersion strategy were conducted for less than 30 min.

The practical benefit of ice clothing lies in its ability to allow athletes to pre-cool during warm-up or while at rest [92]. Ice clothing is typically worn for 15–65 min, which is the optimal time frame for effectively reducing skin temperature [93]. In addition, during warm-ups, whole-body blood flow increases, and the cooling effect of the ice suit aids in distributing cooler blood regions across the body surface [15]. Simultaneously, studies have shown that the mean skin and core temperatures in the intervention group were lower than those in the no-precooling group, suggesting that the vest effectively absorbs excess heat generated during warm-ups [94]. When implementing ice clothing, some studies have found that this strategy can also achieve significant results even when athletes have not warmed up [79]. Uckert et al. [26] reported that ice clothing can directly cool unwarmed muscle tissue, lowering skin temperature until core temperature decreases, thereby enhancing the body’s heat storage capacity or reducing the perception of thermal strain in high-temperature environments. Additionally, cooling inactive muscle tissue can lead to local vasoconstriction [95], altering blood flow redistribution and improving blood delivery to hyperthermic muscle tissue [80]. Marsh et al. [95] also confirmed that ice clothing can effectively reduce heart rate after use in the unwarmed phase.

The head precooling strategy aims to reduce temperature in the head, controlling common carotid artery and vein flow and minimizing excessive heat loss gradient changes [16]. Minett et al. [14] showed that head precooling lowers head temperature prior to exercise, mitigating the impact of a high-temperature environment on athletes’ cognitive function and enhancing endurance exercise performance. In addition, Harris et al. [78] found that perceived changes in thermal comfort are crucial factors in assessing endurance exercise performance in high-temperature environments. However, several studies have shown that head precooling can benefit the stability of the central nervous system in hot environments [36], while thermal comfort during controlled exercise is negatively affected by the hot environment [96].

A previous study has shown that treadmill tests yield superior results compared to cycling in endurance exercise performance assessments, suggesting that equipment type is a crucial factor [97]. Based on this, meta-analysis studies were divided into treadmill and cycling subgroups. Our subgroup analysis revealed that external precooling is more effective in improving TT and TTE performances on treadmills, indicating their superiority for endurance testing [98,99]. Muscle engagement during running exceeds cycling, and treadmills enable superior performance due to full skeletal muscle engagement and self-regulation during exhaustion [100]. Additionally, VO_2_max values are higher on treadmills compared to bicycles under self-paced VO_2_ max tests (SPV), with treadmill exercise yielding more stable physiological and psychological indices [101,102].

Numerous studies in this meta-analysis utilized the SPV method, verifying its reliability in assessing aerobic capacity and resembling competitive performances [103]. Treadmill running engages more muscles and provides exercise substrates more efficiently than cycling, with higher VO_2_ max values [104,105]. Treadmill exercise also stabilizes central system indicators and cognitive function during exercise [106,107]. Recent research highlights the importance of sacral region stability in endurance testing, which treadmills enhance due to their constant speed and environmental constraints [108,109]. Furthermore, treadmills promote aerobic capacity and energy metabolism development and mitigate bone impact during running [110,111,112]. Interestingly, in studies conducted by Katica et al. [32], Chan et al. [33], and Stevens et al. [37], researchers applied fan cooling during endurance exercise performance tests involving bicycles. Consequently, compared to cycling, running may diminish convective air cooling, thereby resulting in a more pronounced effect from precooling. Therefore, treadmills may generally outperform bicycles in endurance exercise performance tests among the general population, while for skilled cyclists, bicycles may outperform treadmills.

### 4.3. Limitations

Several potential limitations of this meta-analysis ought to be acknowledged. Firstly, all the included studies were RCTs of precooling interventions, which inherently precluded complete blinding. Therefore, subjective factors may have influenced the article quality evaluation to some degree. Secondly, the studies included in this meta-analysis solely utilized time as a metric for endurance exercise performance, necessitating caution when extrapolating our findings to related studies focusing on endurance exercise performance. Furthermore, a significant proportion of the included studies involved male subjects, and consequently, this study did not conduct subgroup analysis based on gender. However, the impact of precooling on endurance exercise performance in the heat may be influenced by gender factors. Therefore, future research should further analyze the effects of precooling on endurance exercise performance in different genders, as well as any potential gender differences that may arise. Moreover, the precooling protocols used in the included studies are diverse, so this study only categorizes the precooling strategies into internal and external precooling for analysis. Therefore, this study is currently unable to provide the most effective specific precooling protocol. Finally, the included studies did not undertake subgroup analyses based on factors such as VO_2_max, blood lactate concentration, or pulmonary ventilation. Therefore, future studies may delve deeper into these aforementioned shortcomings.

### 4.4. Practical Implications

The findings of this study offer several practical implications for athletes, coaches, and sports experts involved in endurance sports and training in the heat. Firstly, athletes can gain an advantage by adding precooling strategies to their pre-event preparations when taking part in endurance events in hot conditions. Since external precooling is quite effective, athletes may consider using ice vests, ice jackets, or cold water immersion as part of their warm-up or pre-competition routines. Secondly, coaches and trainers can include precooling in their training plans to help athletes adjust to hot environments and boost their performance in such conditions, especially for running-based endurance events. Lastly, more research is required to understand the long-term impact of precooling on athlete health and performance, and to find the best precooling protocols for different endurance events and environmental conditions.

## 5. Conclusions

Precooling is an effective approach to improve endurance exercise performance in the heat. External precooling is more effective in improving endurance exercise performance, particularly in running-based performance.

## Data Availability

All data generated or analyzed during this study are included in the article/Supplementary Material.

## Supplementary Materials

The following supporting information can be downloaded at: https://www.mdpi.com/article/10.3390/nu16234217/s1, Figure S1: Results of Cochrane risk of bias tool; Figure S2: Funnel plots; Figure S3: Sensitivity analysis results of TT; Figure S4: Sensitivity analysis results of TTE; Table S1: Search strategies; Table S2: Characteristics of studies included in this meta-analysis; Table S3: Strength of outcome evidence.

## Abbreviations

CI, confidence interval; PRISMA, Preferred Reporting Items for Systematic Review and Meta-Analysis; RCTs, randomized controlled trials; RPE, rating of perceived exertion; SD, standard deviation; SE, standard error; SMD, standardized mean difference; SPV, self-paced VO_2_max; TC, thermal comfort; TS, thermal sensation; TT, time trial; TTE, time to exhaustion.
