# Supplementary material for: Effects of Precooling on Endurance Exercise Performance in the Heat: A Systematic Review and Meta-Analysis of Randomized Controlled Trials
Source: Nutrients. 2024 Dec 6;16(23):4217. doi: 10.3390/nu16234217 (PMC11644334; doi:10.3390/nu16234217)

Supplementary Material

Effects of precooling on endurance exercise performance in the heat: a systematic review and meta-analysis of randomized controlled trials

Table S1. Search strategies.. 2

Table S2. Characteristics of studies included in this meta-analysis.. 4

Table S3. Strength of outcome evidence.. 6

Figure S1. Results of Cochrane risk of bias tool.. 7

Figure S2. Funnel plots.. 8

Figure S3. Sensitivity analysis results of TT.. 9

Figure S4. Sensitivity analysis results of TTE.. 10

Table S1. Search c

(1) PubMed

Search number	Query
#1	"exercise"[Title/Abstract] OR "exercising"[Title/Abstract] OR "endurance"[Title/Abstract] OR "performance"[Title/Abstract] OR "pace"[Title/Abstract] OR "pacing"[Title/Abstract] OR "sport"[Title/Abstract] OR "sports"[Title/Abstract] OR "sporting"[Title/Abstract] OR "aerobic"[Title/Abstract]
#2	"precool"[Title/Abstract] OR "pre-cool"[Title/Abstract] OR "pre-cooling"[Title/Abstract] OR "precooling"[Title/Abstract] OR "pre-cooled"[Title/Abstract] OR "precooled"[Title/Abstract] OR "cool"[Title/Abstract] OR "cooled"[Title/Abstract]
#3	#1 AND #2

(2) Web of sciences

Search number	Query
#1	(((((((((TS=("exercise")) OR TS=("exercising")) OR TS=("endurance")) OR TS=("performance")) OR TS=("pace")) OR TS=("pacing")) OR TS=("sport")) OR TS=("sports")) OR TS=("sporting")) OR TS=("aerobic")
#2	(((((((TS=("precool")) OR TS=("pre-cool")) OR TS=("pre-cooling")) OR TS=("precooling")) OR TS=("pre-cooled")) OR TS=("precooled")) OR TS=("cool")) OR TS=("cooled")
#3	#1 AND #2

(3) EBSCO

Search number	Query
#1	TI ("exercise" OR "exercising" OR "endurance" OR "performance" OR "pace" OR "pacing" OR "sport" OR "sports" OR "sporting" OR "aerobic") OR AB ("exercise" OR "exercising" OR "endurance" OR "performance" OR "pace" OR "pacing" OR "sport" OR "sports" OR "sporting" OR "aerobic")

#2	TI ("precool" OR "pre-cool" OR "pre-cooling" OR "precooling" OR "pre-cooled" OR "precooled" OR "cool" OR "cooled") OR AB ("precool" OR "pre-cool" OR "pre-cooling" OR "precooling" OR "pre-cooled" OR "precooled" OR "cool" OR "cooled")
#3	#1 AND #2

(4) Scopus

Search number	Query
#1	TITLE-ABS-KEY("exercise" OR "exercising" OR "endurance" OR "performance" OR "pace" OR "pacing" OR "sport" OR "sports" OR "sporting" OR "aerobic")
#2	TITLE-ABS-KEY("precool" OR "pre-cool" OR "pre-cooling" OR "precooling" OR "pre-cooled" OR "precooled" OR "cool" OR "cooled")
#3	#1 AND #2

(5) Cochrane Library

Search number	Query
#1	("exercise"):ti,ab,kw or ("exercising"):ti,ab,kw or ("endurance"):ti,ab,kw or ("performance"):ti,ab,kw or ("pace"):ti,ab,kw or ("pacing"):ti,ab,kw or ("sport"):ti,ab,kw or ("sports"):ti,ab,kw or ("sporting"):ti,ab,kw or ("aerobic"):ti,ab,kw
#2	("precool"):ti,ab,kw or ("pre-cool"):ti,ab,kw or ("pre-cooling"):ti,ab,kw or ("precooling"):ti,ab,kw or ("pre-cooled"):ti,ab,kw or ("precooled"):ti,ab,kw or ("cool"):ti,ab,kw or ("cooled"):ti,ab,kw
#3	#1 AND #2

Table S2. Characteristics of studies included in this meta-analysis

Study	Environmental conditions (℃)	Sample size	Precooling mode	Precooling protocol	Cooling duration (min)	Performance task	Performance measurement
Arngrımsson et al. (2004)	32	9 males	External	Ice vest	30	Running	TT
	32	8 females	External	Ice vest	30	Running	TT
Coelho et al.(2021)	35	15 males	External	Heah cooling	20	Running	TT
Duffield et al. (2007)	32 ± 1	9 males	External	Ice-bath	15	NR	TT
	32 ± 1	9 males	External	Ice vest	15	NR	TT
Katica et al. (2018)	35.0 ± 0.5	8 males	External	Ice jacket	20	Cycling	TT
Quod et al. (2008)	34.3 ± 1.1	6 males	External	Ice jacket	40	Cycling	TT
	34.3 ± 1.1	6 males	External	Ice water immersion	30	Cycling	TT
Randall et al. (2015)	32.2 ± 0.8	8 males	External	Ice vest	30	Running	TT
	32.2 ± 0.8	8 males	External	Ice jacket	30	Running	TT
Stevens et al. (2016)	33	9 males	External	Ice water immersion	30	Running	TT
Stevens et al. (2017)	32.5 ± 0.1	11 males	Mixed	Combined cold-water immersion and ice slurry ingestion	30	Cycling	TT
	32.5±0.1	11 males	Mixed	Combined facial water spray and menthol mouth rinse	30	Cycling	TT

	32.5 ± 0.1	11 males	Mixed	Combined cold-water immersion, ice slurry ingestion, facial water spray and menthol mouth rinse	30	Cycling	TT
Yeo et al. (2012)	28.2 ± 0.8	8 males and 4 females	Internal	Ice slurry	30	Running	TT
Zimmerman et al. (2017)	34.9 ± 0.3	10 females	Internal	Ice slurry	30	Cycling	TT
Chan et al. (2019)	33	10 males	External	Ice jacket	30	Running	TTE
Iwata et al. (2020)	38	12 males	Internal	Ice slurry	30	Cycling	TTE
	38	12 females	Internal	Ice slurry	30	Cycling	TTE
Nakamura et al. (2020)	35	8 males	Internal	Ice water immersion	15	Cycling	TTE
	35	8 males	External	Ice slurry	15	Cycling	TTE
Siegel et al.(2012)	34.0 ± 0.1	8 males	External	Ice water immersion	30	Running	TTE
	34.0 ± 0.1	8 males	Internal	Ice slurry	30	Running	TTE
Uckert et al. (2007)	31	20 males	External	Ice vest	20	Running	TTE

Abbreviations: TT, time trial; TTE, time to exhaustion.

Table S3. Strength of outcome evidence

Quality assessment							No of patients		Effect	Quality	Importance
No of studies	Design	Risk of bias	Inconsistency	Indirectness	Imprecision	Other considerations	Experimental	Control	Absolute effect (95% CI)		
Time to exhaustion (Better indicated by lower values)											
16	randomized trials	no serious risk of bias	no serious inconsistency	no serious indirectness	no serious imprecision	reporting bias[1]	150	150	SMD: -0.37 lower (0.6 to 0.14 lower)	ÅÅÅO MODERATE	CRITICAL
Time trial (Better indicated by lower values)											
8	randomized trials	serious[2]	serious[3]	no serious indirectness	no serious imprecision	reporting bias[1]	86	86	SMD: -0.73 higher (0.41 to 1.05 higher)	ÅÅOO LOW	CRITICAL

SMD, standardized mean difference; CI, Confidence interval.

[1] The funnel plot is asymmetric, indicating the potential presence of publication bias.

[2] Two studies exhibit a high risk of detection bias.

[3] The I-squared aggregated by the time trial indicator is 50%.

Figure S1. Results of Cochrane risk of bias tool

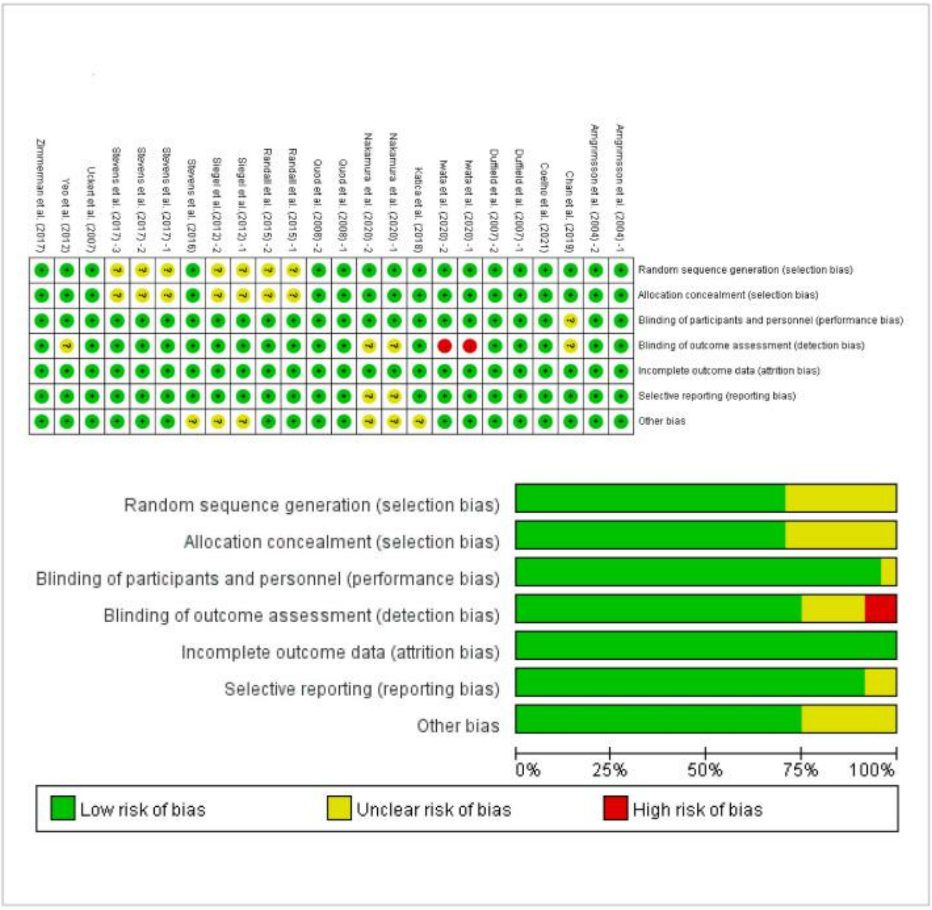

Figure S2. Funnel plots

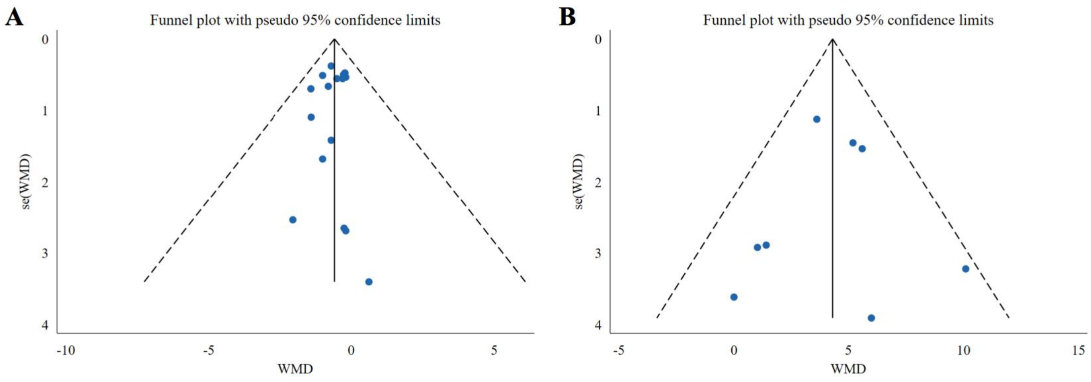

A. TT; **B.** TTE.

Figure S3. Sensitivity analysis results of TT

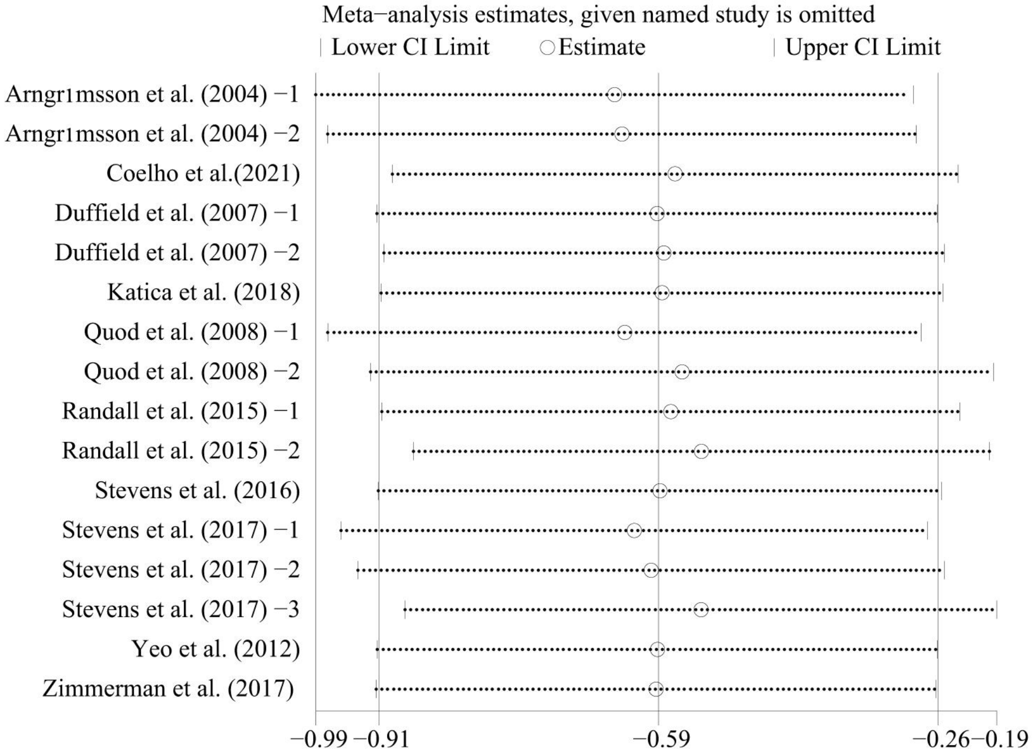

Figure S4. Sensitivity analysis results of TTE

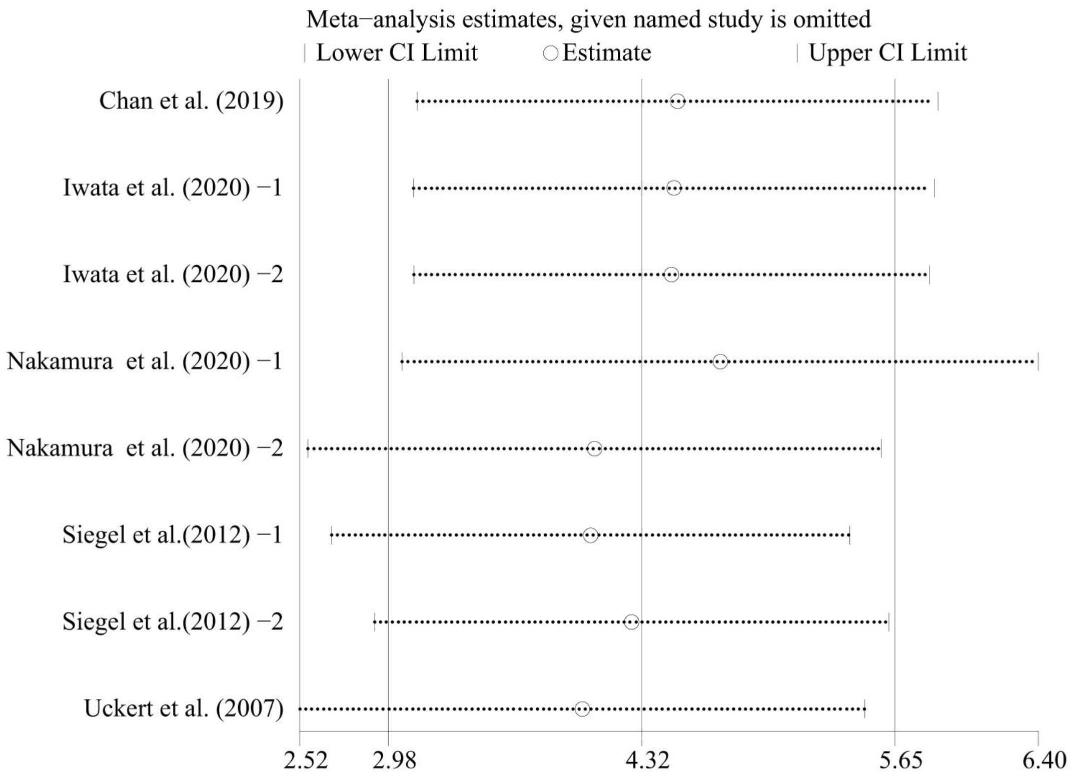

Supplement: Supplementary file 1 [file nutrients-16-04217-s001.zip › nutrients-3354899-supplementary.pdf]
